# Efficacy of Erector Spinae Nerve Block for Pain Control After Spinal Surgeries: An Updated Systematic Review and Meta-Analysis

**DOI:** 10.3389/fsurg.2022.845125

**Published:** 2022-02-28

**Authors:** Mingda Duan, Yuhai Xu, Qiang Fu

**Affiliations:** ^1^Department of Anesthesiology, Hainan Hospital of General Hospital of PLA, Sanya, China; ^2^Department of Anesthesiology, Air Force Medical Center, Beijing, China; ^3^Department of Anesthesiology, The First Medical Center of General Hospital of PLA, Beijing, China

**Keywords:** erector spinae plane block, spinal surgery, post-operative analgesia, complications, meta-analysis

## Abstract

**Background:**

Erector spinae plane block (ESPB), as a regional anesthesia modality, is gaining interest and has been used in abdominal, thoracic and breast surgeries. The evidence on the efficacy of this block in spinal surgeries is equivocal. Recently published reviews on this issue have concerning limitations in methodology.

**Methods:**

A systematic search was conducted using the PubMed, Scopus, Embase, and Cochrane Central Register of Controlled Trials (CENTRAL). Randomized controlled trials (RCTs) that were done in patients undergoing spinal surgery and had compared outcomes of interest among those that received ESPB and those with no block/placebo were considered for inclusion. Statistical analysis was performed using STATA software. GRADE assessment was done for the quality of pooled evidence.

**Results:**

A total of 13 studies were included. Patients receiving ESPB had significantly reduced total opioid use (Standardized mean difference, SMD −2.76, 95% CI: −3.69, −1.82), need for rescue analgesia (Relative risk, RR 0.38, 95% CI: 0.22, 0.66) and amount of rescue analgesia (SMD −5.08, 95% CI: −7.95, −2.21). Patients receiving ESPB reported comparatively lesser pain score at 1 h (WMD −1.62, 95% CI: −2.55, −0.69), 6 h (WMD −1.10, 95% CI: −1.45, −0.75), 12 h (WMD −0.78, 95% CI: −1.23, −0.32) and 24 h (WMD −0.54, 95% CI: −0.83, −0.25) post-operatively. The risk of postoperative nausea and vomiting (PONV) (RR 0.32, 95% CI: 0.19, 0.54) was lower in those receiving ESPB. There were no differences in the duration of surgery, intra-operative blood loss and length of hospital stay between the two groups. The quality of pooled findings was judged to be low to moderate.

**Conclusions:**

ESPB may be effective in patients with spinal surgery in reducing post-operative pain as well as need for rescue analgesic and total opioid use. In view of the low to moderate quality of evidence, more trials are needed to confirm these findings.

**Systematic Review Registration:** http://www.crd.york.ac.uk/PROSPERO/, identifier: CRD42021278133.

## Introduction

One of the ensuing complications of spinal surgery is the post-operative pain that could range from moderate to severe and could have an impact on timing of mobilization and rehabilitation, length of hospital stay, patient satisfaction and could lead to chronic backpain (1–3). Delayed mobilization could also increase the risk of thrombo-embolic events, delayed wound healing and nosocomial infections (4, 5). The management of post-operative pain in spinal surgeries has been a challenge. The conventional analgesia model is based on use of opioids and therefore, the opioid- related side effects cannot be avoided (6). These side effects include nausea, vomiting, pruritis, urinary retention and dizziness and could be worrisome for the patients (6). One of the techniques to reduce post-operative pain and opioid related side effects is multimodal analgesic (MMA) regimen (7). It involves use of a variety of drugs and delivery mechanisms.

Use of regional anesthesia is an important component of MMA (7, 8). Recently, a novel method for regional anesthesia named erector spinae plane block (ESPB) has received wide attention. Initially the use of ESPB was demonstrated for treating pain associated with shingles (9). It is a paravertebral interfascial block wherein the anesthetic is injected between the transverse process of the vertebrae and erector spinae muscle (10, 11). Upon being injected, the anesthetic diffuses both cranially and caudally and exerts its action on the ventral and dorsal rami of the spinal nerves (9–11). As the local anesthetic is injected in a plane that is farther from the spinal cord, the associated risk of causing trauma to the cord and consequent complication is minimized. Also, the block is relatively easier to perform and is done under the guidance of ultrasonography. Due to all its features, the use of ESPB is common in abdominal such as laparoscopic cholecystectomy, breast and thoracic surgery (12–14). The role of ESPB in spinal surgeries is being explored through recent randomized controlled trials (RCTs); however, the results are conflicting. There have been systematic reviews and meta-analyses published on this issue recently but the methodology in most of these reviews is not adequate.

A systematic review by Jun Ma et al. included 12 studies with 828 patients and documented that ESPB reduced postoperative pain scores and significantly decreased opioid consumption, compared to no block/placebo (15). Further a reduced the incidence of rescue analgesia and postoperative nausea and vomiting (PONV) was noted. A major shortcoming of the review is that the authors majorly included research outputs that were non-peer reviewed (i.e., thesis/dissertation). Another review by Liu et al. found that ESPB significantly reduced postoperative opioid consumption, pain scores within 24 h post-operative period, need for rescue analgesia and risk of PONV (16). However, some studies that were eligible to be included in the review were not included. Further, there have been recent publication of several new studies on this aspect. Taking into account these considerations, there is a need for a comprehensive, methodologically robust systematic review and meta-analysis to assess the efficacy of ESPB, compared to placebo or no block for patients undergoing spinal surgical procedures. The underlying hypothesis for this meta-analysis was that erector spinae block in patients undergoing spinal surgery will lead to better pain control, reduced need for rescue analgesia, reduced need for opioid and lower risk of complications, compared to placebo or no block.

## Materials and Methods

### Search Strategy

PubMed, Scopus, Embase, and Cochrane Central Register of Controlled Trials (CENTRAL) databases were systematically searched for English language papers published prior to 15th September 2021 using medical subject heading (MeSH) terminology and free text words (Supplementary Table 1). This meta-analysis was conducted in compliance with PRISMA (Preferred Reporting Items for Systematic Reviews and Meta-analyses) guidelines (17) and is registered in the International Prospective Registry of Systematic Reviews (PROSPERO; registration number-CRD42021278133). The literature search aimed at identifying studies that examined the outcomes of interest between patient groups receiving erector spinae plane block (ESPB) and those that received either no block or placebo for spinal surgery. To elaborate further, the population of interest were subjects undergoing spinal surgery, intervention was the use of erector spinae plane block, comparator was the use of placebo or no block and primary outcomes of interest were post-operative patient reported pain scores, total opioid use post-operatively, need for rescue analgesia and risk of complications. Only studies that adopted a randomized controlled design were of interest for the meta-analysis.

### Selection Criteria and Methods

After duplicate removal, two subject experts reviewed all studies identified by the search strategy. First, titles and abstracts were examined, followed by subsequent full text review. Any differences of opinion on study inclusion suitability were resolved through discussion. Only those studies were included in the meta-analysis that fulfilled the inclusion criteria. In order to identify additional literature, the reference list of the included studies was also reviewed.

#### Inclusion Criteria

Randomized controlled trials (RCTs) that were done in patients undergoing spinal surgery and had compared outcomes of interest among those that received ESPB and those with no block/placebo were considered for inclusion.

#### Exclusion Criteria

Observational studies, case studies, and reviews were excluded. Studies not providing data on the outcomes of interest or not providing comparative findings based on ESPB and placebo/no block were excluded.

### Data Extraction and Quality Assessment

Two separate individuals individually extracted relevant data from included studies using a pre-established data extraction sheet. Data extracted included study identifiers (author names, research year), study settings, study design, subject characteristics, procedure performed, anesthetic dosage, sample size, and main findings. Study quality was assessed using the Cochrane risk of bias assessment tool (18).

### Statistical Analysis

This meta-analysis was conducted using STATA version 16.0 and reported effect sizes as pooled relative risks (RR), weighted mean differences (WMD), or standardized mean differences (SMD). WMD was reported for continuous outcomes using the same units whereas SMD for continuous outcomes with different units. Continuous data presented in studies as medians with ranges were converted to means with standard deviations according to a method described by Hozo et al. (19). All effect sizes were reported along with 95% confidence intervals (CI). *I*^2^ indicated heterogeneity, and when *I*^2^ exceeded 40%, a random effects model was used (20). *P*-values of < 0.05 were considered statistically significant. Egger's test was used to assess publication bias (21). The quality of evidence generated for the outcomes considered was assessed through GRADE criteria using GRADEpro software (22, 23).

## Results

### Selection of Articles, Study Characteristics, and Study Quality

**Figure 1:**
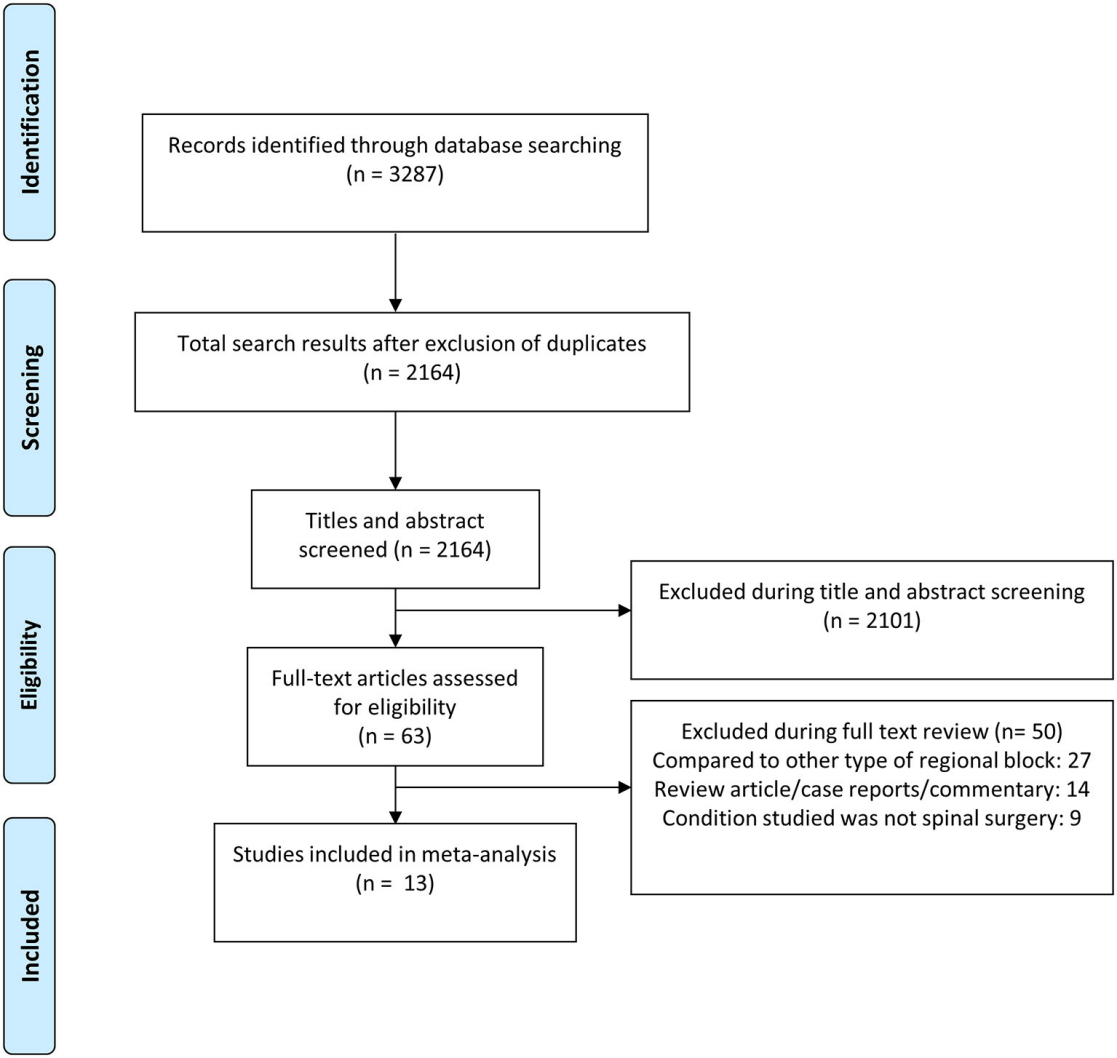
Selection process of the studies included in the review.

The search strategy yielded 2,164 unique citations (Figure 1). Title and abstract screening eliminated 2,101 citations. Out of the remaining 63 studies, 50 were excluded after full-text review, leaving 13 studies for inclusion (Table 1) (24–36). All the included studies were RCTs. Five studies each were done in China and Turkey. Two studies were done in India and remaining one in Ireland. The included studies were of modest to good quality (Supplementary Table 2). All 13 studies reported random sequence generation. In 10 out of 13 studies, allocation concealment was done and in remaining 3, it is unclear whether it was done. Blinding of participants and personnel was done in 6 studies. In all the studies blinding of outcome assessment was done. GRADE assessment was done for the quality of pooled evidence. The findings of the GRADE assessment are presented as Supplementary Table 3.

**Table 1:** Characteristics and key findings of the studies included in the meta-analysis.

**Author (year of publication)**	**Study design**	**Country**	**Participant characteristics including procedure done**	**Dose (for each side)**	**Comparator groups**	**Sample size**	**Key outcomes (compared to no block)**
Yu et al. (24)	RCT	China	Patients of lumbar fracture undergoing posterior lumbar spinal surgery; mean age of around 55 years; majority female (55%)	Intervention: 30 mL of 0.25% Bupivacaine Control: Normal saline	Ultrasound guided lumbar erector spinae plane (ESP) block vs. no block	80 (40 each in intervention and control)	**Length of hospital stay (days); mean (SD**): 12.38 (0.32) vs. 14.78 (0.33) **Duration of surgery (hours); mean (SD**): 1.37 (0.30) vs. 1.41 (0.30) **Numeric pain rating scale (NRS) at rest** 6 h: 0 (0) vs. 1 (0.5) 12 h: 0 (0) vs. 1 (0.25) 24 h: 0 (0.25) vs. 1 (0.5) 48 h: 1 (0.5) vs. 1 (0.5) **Numeric pain rating scale (NRS) on movement** 6 h: 0 (0) vs. 2 (0.5) 12 h: 0 (0.25) vs. 3 (0.5) 24 h: 1 (0) vs. 2.5 (0.44) 48 h: 2 (0.5) vs. 2 (0.75) **Dose of rescue analgesic (Pethidine, mg):** 96.25 (13.68) vs. 245 (13.13) **Total opioid consumption (mg); mean (SD):** 152 (11) vs. 222 (10.75) *Complications* **Postoperative nausea and vomiting**: RR 0.21 (95% CI: 0.11, 0.42) **Pruritis**: RR 0.42 (95% CI: 0.16, 1.07) **Chronic postoperative pain** (pain for atleast 3 months post-operatively): RR 0.50 (95% CI: 0.17, 1.53)
Zhu et al. (25)	RCT	China	Patients undergoing posterior lumbar fusion surgery; mean age of around 60 years; majority female (63%)	Intervention: 20 mL of 0.375% Ropivacaine Control: Normal saline	Ultrasound guided lumbar erector spinae plane (ESP) block vs. no block	40 (20 each in intervention and control)	**Duration of surgery (hours); mean (SD**): 2.13 (0.1) vs. 2.05 (0.08) **Visual analogue scale (VAS) score at rest** 6 h: 0.5 (0.25) vs. 2 (0) 12 h: 0 (0.25) vs. 2 (0) 24 h: 1 (0.25) vs. 2 (0.25) 48 h: 0 (0) vs. 0 (0.25) **VAS score on movement** 6 h: 1.5 (0.25) vs. 3 (0) 12 h: 1 (0.25) vs. 3 (0) 24 h: 2 (0.5) vs. 3 (0.25) 48 h: 1 (0) vs. 1 (0.25) **Need for rescue analgesic (Sufentanil):** RR 0.20 (95% CI: 0.05, 0.80) **Total opioid consumption (mg); mean (SD):** 23.10 (3.1) vs. 36.40 (4.2) *Complications* **Abdominal bloating**: RR 0.83 (95% CI: 0.30, 2.29) **Dizziness**: RR 0.67 (95% CI: 0.13, 3.57)
Yörükoglu et al. (26)	RCT	Turkey	Patients with lumbar disc prolapse undergoing elective single level lumbar microdisectomy; mean age of around 50 yrs	Intervention: 20 mL of 0.25% Bupivacaine Control: Normal saline	Ultrasound guided lumbar erector spinae plane (ESP) block vs. no block	54 (28 in intervention and 26 in control)	**Duration of surgery (hours); mean (SD**): 2.34 (0.52) vs. 2.25 (0.51) **Numeric pain rating scale (NRS) score at rest** 1 h: 3.6 (2.6) vs. 3.5 (2.5) 6 h: 1.71 (1.3) vs. 1.5 (1.4) 12 h: 1.2 (1.2) vs. 1.2 (1.2) 24 h: 1.1 (1.2) vs. 1.5 (1.7) **Need for rescue analgesic:** RR 0.33 (95% CI: 0.14, 0.79)
							**Total opioid consumption (morphine, mg); mean (SD):** 11.3 (9.5) vs. 27 (16.7) *Complications* **Postoperative nausea and vomiting**: RR 0.29 (95% CI: 0.11, 0.76)
Goel et al. (27)	RCT	India	Patients undergoing single level lumbar spine fusion surgery; mean age of around 50 yrs; 58% female	Intervention: 20 mL of 0.25% Bupivacaine Control: Normal saline	Ultrasound guided lumbar erector spinae plane (ESP) block vs. conventional opioid based multimodal post-operative analgesia	101 (51 in intervention and 50 in control)	**Duration of surgery (hours); mean (SD**): 2.19 (0.19) vs. 2.22 (0.20) **Numeric pain rating scale (NRS) score at rest** 0–1 h: 1.52 (1.03) vs. 4.08 (1.78) 6 h: 1.92 (0.84) vs. 2.28 (0.70) 12 h: 1.78 (0.81) vs. 2.1 (0.78) 24 h: 1.09 (0.64) vs. 1.46 (0.68) 48 h: 0.57 (0.57) vs. 0.74 (0.63) **Total opioid use (Fentanyl, mcg)**, **mean (SD):** 100.98 (15.15) vs. 158 (23.38) **Intraoperative blood loss (ml); mean (SD):** 305.88 (88.12) vs. 437 (116.85) *Complications* **Postoperative nausea and vomiting**: RR 0.09 (95% CI: 0.01, 0.66) **Pruritis**: RR 0.09 (95% CI: 0.01, 1.57)
Yesiltas et al. (28)	RCT	Turkey	Patients undergoing posterior spinal instrumentation and fusion for spondylolisthesis; mean age of around 60 yrs; 68% female	Intervention: 20 mL of 0.25% Bupivacaine Control: Normal saline	Ultrasound guided lumbar erector spinae plane (ESP) block vs. no block	56 (28 each in intervention and control)	**Length of hospital stay (days); mean (SD**): 1.71 (0.76) vs. 3.3 (0.98) **Duration of surgery (hours); mean (SD**): 4.38 (1.35) vs. 4.08 (1.62) **VAS score at rest** 1 h: 2.7 (1.18) vs. 4.2 (1.4) 6 h: 2.3 (1.08) vs. 3.3 (1.3) 12 h: 2.1 (1.29) vs. 2.9 (1.1) 24 h: 2.04 (1.14) vs. 2.5 (1.2) **VAS score at movement** 24 h: 2.1 (0.84) vs. 4.1 (0.98) **Total opioid use (morphine, mg)**, **mean (SD):** 33.75 (6.81) vs. 44.75 (12.3) **Need for rescue analgesic:** RR 0.27 (95% CI: 0.09, 0.87) **1st analgesic demand time (min); mean (SD):** 342.6 (58.6) vs. 192.2 (41.8) *Complications* **Postoperative nausea and vomiting**: RR 1.40 (95% CI: 0.50, 3.89)
Zhang et al. (29)	RCT	China	Patients undergoing lumbar spinal fusion surgery; mean age of around 60 yrs; 75% male	Intervention: 20 mL of 0.4% Ropivacaine Control: Normal saline	Ultrasound guided lumbar erector spinae plane (ESP) block vs. no block	60 (30 each in intervention and control)	**Length of hospital stay (days); mean (SD**): 8.0 (0.5) vs. 8.0 (0.5) **Duration of surgery (hours); mean (SD**): 2.54 (0.65) vs. 2.39 (0.56) **NRS score at rest** 6 h: 1.4 (0.5) vs. 2.75 (4.8) 12 h: 1.4 (0.4) vs. 2.1 (3.7) 24 h: 1.3 (0.20) vs. 1.5 (2.1) 48 h: 1.0 (0.15) vs. 1.3 (2.3) **NRS score at movement** 6 h: 2.7 (1.3) vs. 3.5 (5.1) 12 h: 3.25 (2.2) vs. 3.75 (4.7)
							24 h: 3.10 (1.5) vs. 3.23 (4.75) 48 h: 2.9 (1.5) vs. 3.2 (4.8) **Dose of rescue analgesic (Sufentanil, mcg):** 22.5 (7.65) vs. 23.3 (10.95) **Need for rescue analgesic:** RR 0.96 (95% CI: 0.86, 1.08) **Intraoperative blood loss (ml); mean (SD):** 300 (53.2) vs. 275 (25.0) *Complications* **Postoperative nausea and vomiting**: RR 0.33 (95% CI: 0.07, 1.52) **Dizziness:** RR 0.67 (95% CI: 0.12, 3.71)
Finnerty et al. (30)	RCT	Ireland	Patients undergoing thoracolumbar decompression spinal surgery; mean age of around 60 yrs; 50% male	Intervention: 40 mL of 0.25% levobupivacaine Control: None; normal saline not provided	Ultrasound guided lumbar erector spinae plane (ESP) block vs. no block	60 (30 each in intervention and control)	**Duration of surgery (hours); mean (SD**): 3.4 (1.2) vs. 3.2 (1.2) **Verbal response score (VRS) at rest** 12 h: 2.1 (1.9) vs. 3.5 (2.6) 24 h: 2.5 (2.2) vs. 2.6 (1.9) **VRS score at movement** 12 h: 2.5 (3.8) vs. 5.6 (2.5) 24 h: 4.5 (2.7) vs. 5.1 (2.3) **Total opioid use (oxycodone, mg)**, **mean (SD):** 19.4 (25.8) vs. 26.8 (18.4)
Zhang et al. (31)	RCT	China	Patients undergoing lumbar spinal surgery; mean age of around 61 yrs; 66% female	Intervention: 25 mL of 0.3% ropivacaine Control: None	Ultrasound guided lumbar erector spinae plane (ESP) block vs. conventional opioid-based multimodal analgesia	59 (30 in intervention and 29 in control)	**NRS score at rest** 24 h: 1.0 (0.25) vs. 2.0 (0.5) 48 h: 1.0 (0.25) vs. 1.0 (0.5) **NRS score at movement** 24 h: 3.0 (0.5) vs. 3.0 (0.75) 48 h: 2.0 (0.25) vs. 3.0 (0.5) **Total opioid use (mg)**, **mean (SD):** 56 (5.1) vs. 61 (5.75) **1st analgesic demand time (min); mean (SD):** 480 (187.8) vs. 60 (82.2)
Yayik et al. (32)	RCT	Turkey	Patients undergoing lumbar spinal decompression surgery; mean age of around 50 yrs; 60% male	Intervention: 20 mL of 0.025% bupivacaine Control: None	Ultrasound guided lumbar erector spinae plane (ESP) block vs. no block	60 (30 each in intervention and control)	**Duration of surgery (hours); mean (SD**): 1.52 (0.54) vs. 1.48 (0.33) **VAS score at rest** 1 h: 1.10 (1.03) vs. 3.70 (1.60) 6 h: 1.93 (0.87) vs. 3.83 (1.18) 12 h: 2.40 (0.89) vs. 3.37 (1.35) 24 h: 2.00 (1.36) vs. 2.83 (1.51) **VAS score at movement** 1 h: 1.53 (1.04) vs. 4.20 (1.40). 6 h: 2.30 (0.60) vs. 4.63 (1.10) 12 h: 2.63 (0.56) vs. 3.77 (0.82) 24 h: 2.30 (1.06) vs. 3.23 (0.77) **Total opioid consumption (mg); mean (SD):** 268.33 (71.44) vs. 370.33 (73.27) **Need for rescue analgesic:** RR 0.30 (95% CI: 0.09, 0.98) **1st analgesic demand time (min); mean (SD):** 325.17 (22.82) vs. 174.17 (22.82) *Complications* **Postoperative nausea and vomiting**: RR 0.29 (95% CI: 0.06, 1.26)
Eskin et al. (33)	RCT	Turkey	Patients undergoing elective lumbar decompression surgery; mean age of around 58 yrs; 60% female	Intervention: 20 mL of 0.25% bupivacaine Control: None	Ultrasound guided lumbar erector spinae plane (ESP) block vs. no block	80 (40 each in intervention and control)	**Duration of surgery (hours); mean (SD**): 2.07 (0.15) vs. 2.14 (0.13) **VAS score at rest** 1 h: 1.6 (0.5) vs. 4.1 (1.7) 6 h: 1.4 (0.8) vs. 3.6 (1.3) 12 h: 1.9 (0.5) vs. 3.5 (0.9) 24 h: 2.2 (0.1) vs. 2.9 (0.8) 48 h: 1.9 (0.9) vs. 2.0 (0.1) **Total opioid consumption (mg); mean (SD):** 254.1 (11.2) vs. 370.7 (23.6) **Dose of rescue analgesic (mg):** 10 (0.9) vs. 44.1 (4.17) **Need for rescue analgesic:** RR 0.21 (95% CI: 0.10, 0.41) **1st analgesic demand time (min); mean (SD):** 852 (96) vs. 18 (6) *Complications* **Postoperative nausea and vomiting**: RR 0.14 (95% CI: 0.02, 1.11) **Pruritis**: RR 0.17 (95% CI: 0.02, 1.32)
Ciftci et al. (34)	RCT	Turkey	Patients undergoing single level lumbar discectomy and hemilaminectomy; mean age of around 45 yrs; 50% males	Intervention: 20 mL of 0.25% bupivacaine Control: None	Ultrasound guided lumbar erector spinae plane (ESP) block vs. no block	60 (30 each in intervention and control)	**Duration of surgery (hours); mean (SD**): 1.19 (0.28) vs. 1.28 (0.34) **VAS score at rest** 1 h: 1 (0.75) vs. 3 (0.5) 6 h: 1 (0.5) vs. 2 (0.75) 12 h: 1 (1) vs. 1 (0.5) 24 h: 0 (0.25) vs. 0 (0.25) **VAS score at movement** 1 h: 2 (1) vs. 4 (0.5) 6 h: 2 (0.5) vs. 3 (0.75) 12 h: 1 (1.25) vs. 2 (0.75) 24 h: 0 (0.75) vs. 1 (0.5) **Total opioid consumption (mcg); mean (SD):** 250 (56.3) vs. 375 (76.2) **Dose of rescue analgesic (mcg):** 20 (35) vs. 140 (45) **Need for rescue analgesic:** RR 0.43 (95% CI: 0.24, 0.78) *Complications* **Postoperative nausea and vomiting**: RR 0.25 (95% CI: 0.11, 0.58) **Pruritis**: RR 1.75 (95% CI: 0.57, 5.36)
Singh et al. (35)	RCT	India	Patients scheduled to undergo elective lumbar spine surgery (prolapsed lumbar intervertebral disk, lumbar stenosis, or laminectomy); mean age of 35 years; 85% males	Intervention: 20 mL of 0.25% bupivacaine Control: None	Intervention group- Ultrasound guided lumbar erector spinae plane (ESP) Control group-standard analgesia with no preoperative ESP block Both groups received standard general anesthesia	40 (20 each in intervention and control)	**Duration of surgery (hours); mean (SD**): 2.49 (0.11) vs. 2.42 (0.13) **NRS score at rest** 1 h: 2 (0.5) vs. 2 (0.5) 6 h: 4 (0.75) vs. 5 (0.75) 12 h: 2 (0.5) vs. 2 (0.5) 24 h: 2 (0.5) vs. 2 (0.5) **Total opioid consumption (mg); mean (SD):** 1.4 (1.5) vs. 7.2 (2.0) **Need for rescue analgesic:** RR 0.45 (95% CI: 0.28, 0.73) **1st analgesic demand time (min); mean (SD):** 348 (45) vs. 144 (35.4) *Complications* **Postoperative nausea and vomiting**: RR 0.20 (95% CI: 0.01, 3.92)
Zhang et al. (36)	RCT	China	Patients scheduled to undergo open posterior lumbar decompression surgery; mean age of 64 years; 65% females	Intervention: 25 mL of 0.3% ropivacaine Control: None	Intervention group- Ultrasound guided lumbar erector spinae plane (ESP) Control group-no block	60 (30 each in intervention and control)	**Duration of surgery (hours); mean (SD**): 3.17 (0.95) vs. 3.45 (0.97) **Length of hospital stay (days); mean (SD**): 6 (1.07) vs. 6.5 (1.33) **NRS score at rest** 24 h: 1.5 (1.1) vs. 2.1 (1.5) 48 h: 0.9 (0.7) vs. 1.6 (1.3) **NRS score at movement** 24 h: 2.9 (1.4) vs. 3.7 (1.9) 48 h: 2.0 (0.9) vs. 3.0 (1.6) **Total opioid consumption (mg); mean (SD):** 9.1 (2.1) vs. 21.8 (3.4) **Dose of rescue analgesic (Sufentanil, mcg):** 20 (7) vs. 25 (5.6) **1st analgesic demand time (min); mean (SD):** 570 (140) vs. 60 (90) *Complications* **Postoperative nausea and vomiting**: RR 2.0 (95% CI: 0.19, 20.9)

### Postoperative Opioid Consumption and Need for Rescue Analgesia

**Figure 2:**
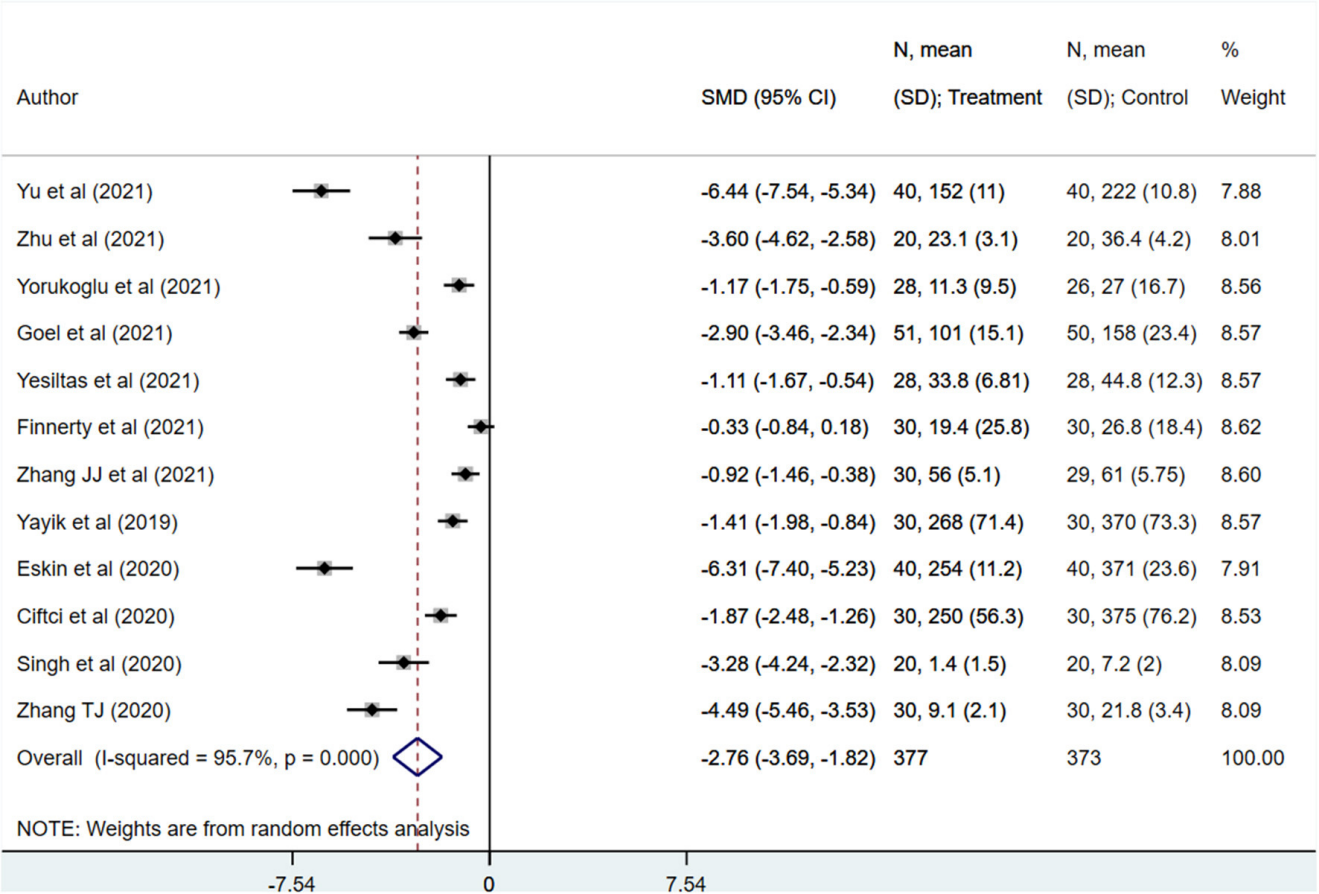
Comparison of postoperative opioid consumption among subjects with erector spinae plane block, compared to placebo or no block.

**Figure 3:**
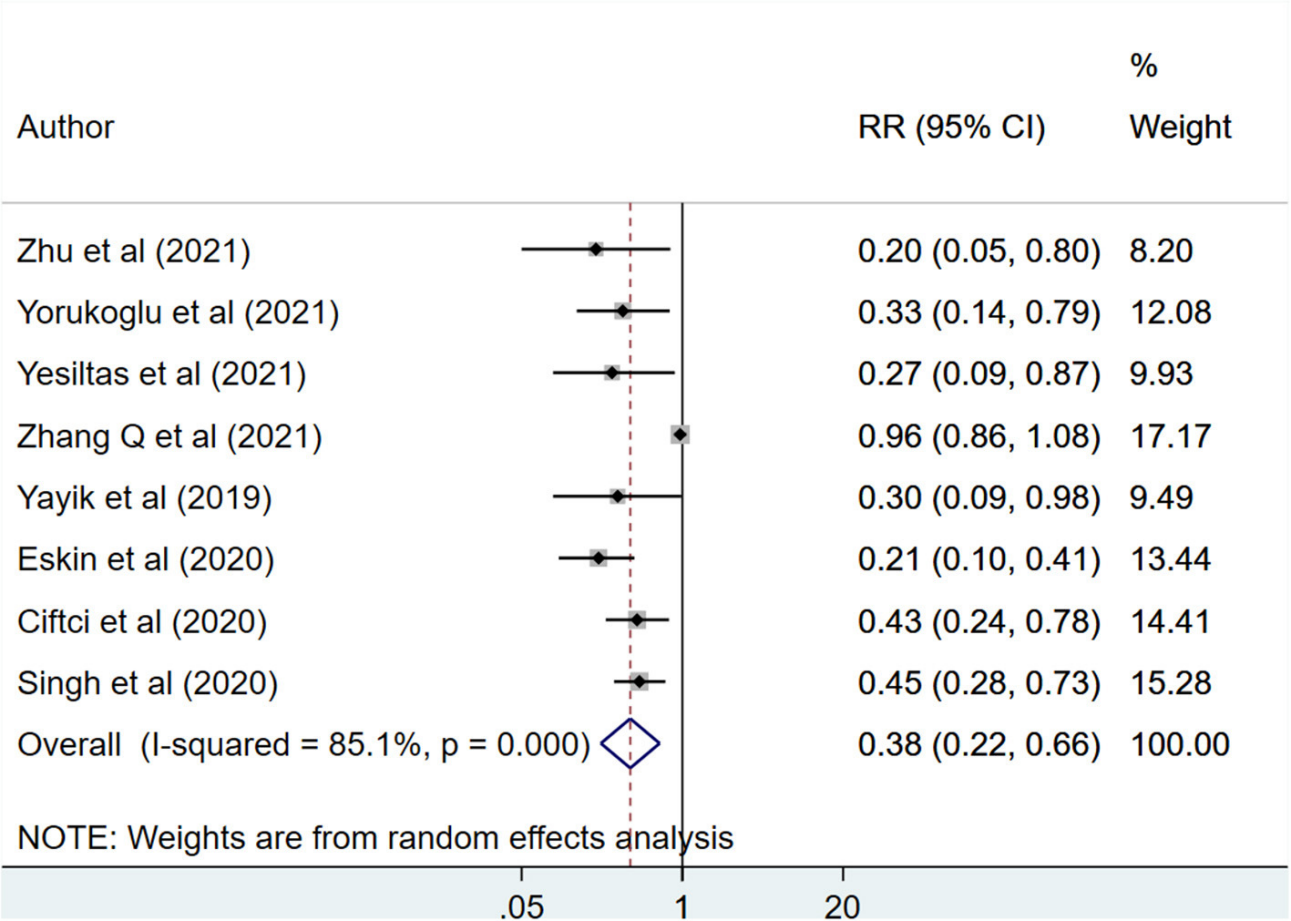
Risk for need of rescue analgesia among subjects with erector spinae plane block, compared to placebo or no block.

In patients that received erector spinae plane block (ESPB), the total opioid use in the first 48 h post-surgery was significantly lower (SMD −2.76, 95% CI: −3.69, −1.82; *N* = 12, *I*^2^ = 95.7%) than patients with placebo/no block (Figure 2). Egger's test indicated no evidence of publication bias (*P* = 0.34). Compared to patients with placebo/no block, those with ESPB had significantly reduced need for rescue analgesia (RR 0.38, 95% CI: 0.22, 0.66; *N* = 8, *I*^2^ = 85.1%) (Figure 3). Egger's test indicated no evidence of publication bias (*P* = 0.18). Also, those receiving ESPB required lower amount of analgesia (SMD −5.08, 95% CI: −7.95, −2.21; *N* = 5, *I*^2^ = 98.5%) and had a higher demand time (in minutes) (WMD 377.7, 95% CI: 163.51, 591.99; *N* = 6, *I*^2^ = 99.7%) (Table 2).

**Table 2:** Summary of pooled findings related to additional outcomes of interest.

**Outcome**	**Pooled effect size (95% confidence interval, CI)**	**Number of studies (no. of participants)**	***I*** **^2^ (%)**
Duration of surgery (hours)	WMD 0.01 (−0.05, 0.06)	12 (*n =* 751)	49.0
Length of hospital stay (days)	WMD −1.13 (−2.55, 0.29)	4 (*n =* 256)	98.9
Dose of rescue analgesic	SMD −5.08 (−7.95, −2.21)*	5 (*n =* 340)	98.5
1st analgesic demand time (min)	WMD 377.7 (163.51, 591.99)*	6 (*n =* 355)	99.7
Intraoperative blood loss (ml)	WMD −52.51 (−206.36, 101.33)	2 (*n =* 161)	97.8

* *Statistically significant at P < 0.05*.

### Post-operative Pain Scores on Rest and Movement

**Figure 4:**
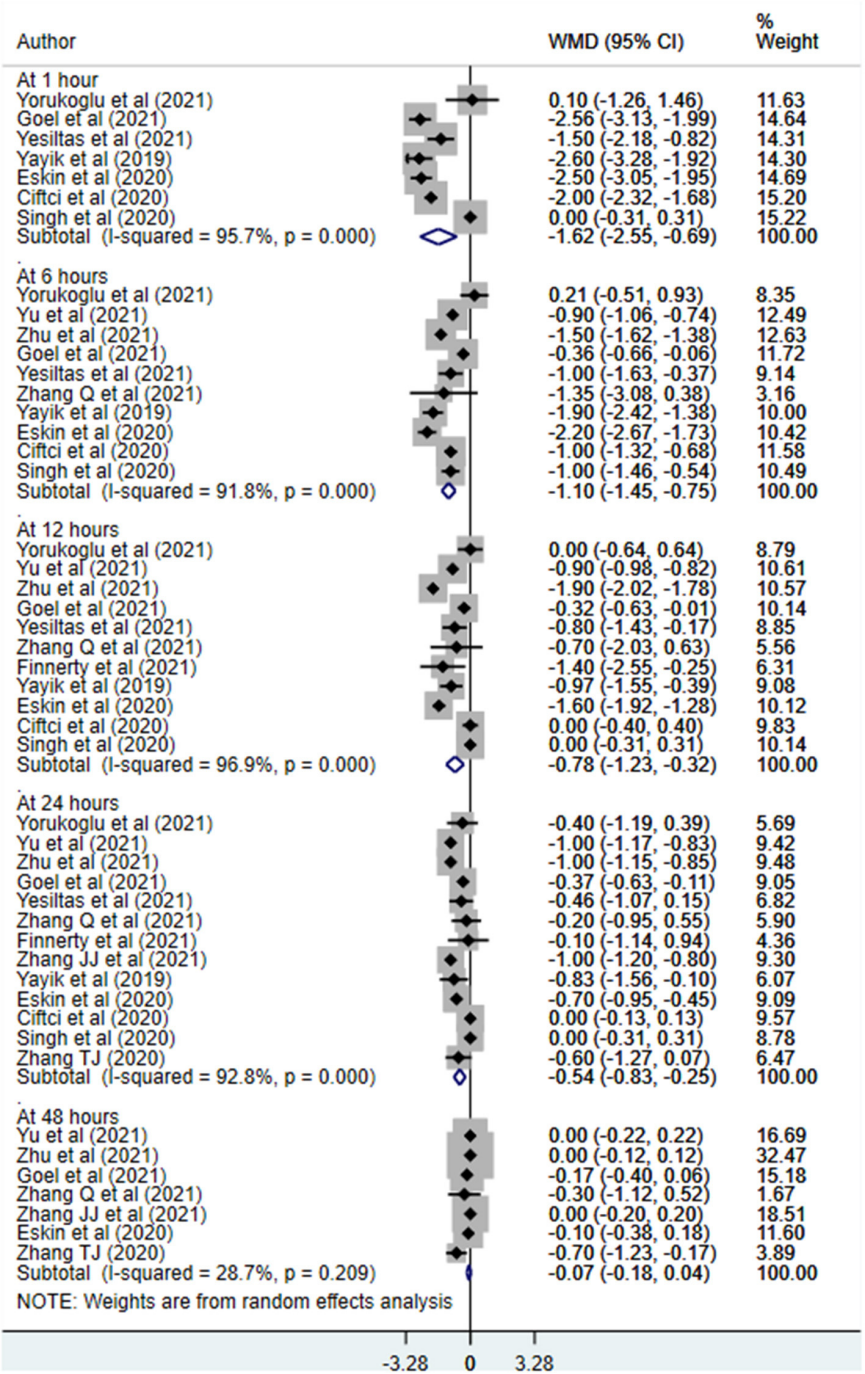
Postoperative pain scores at rest among subjects with erector spinae plane block, compared to placebo or no block.

**Figure 5:**
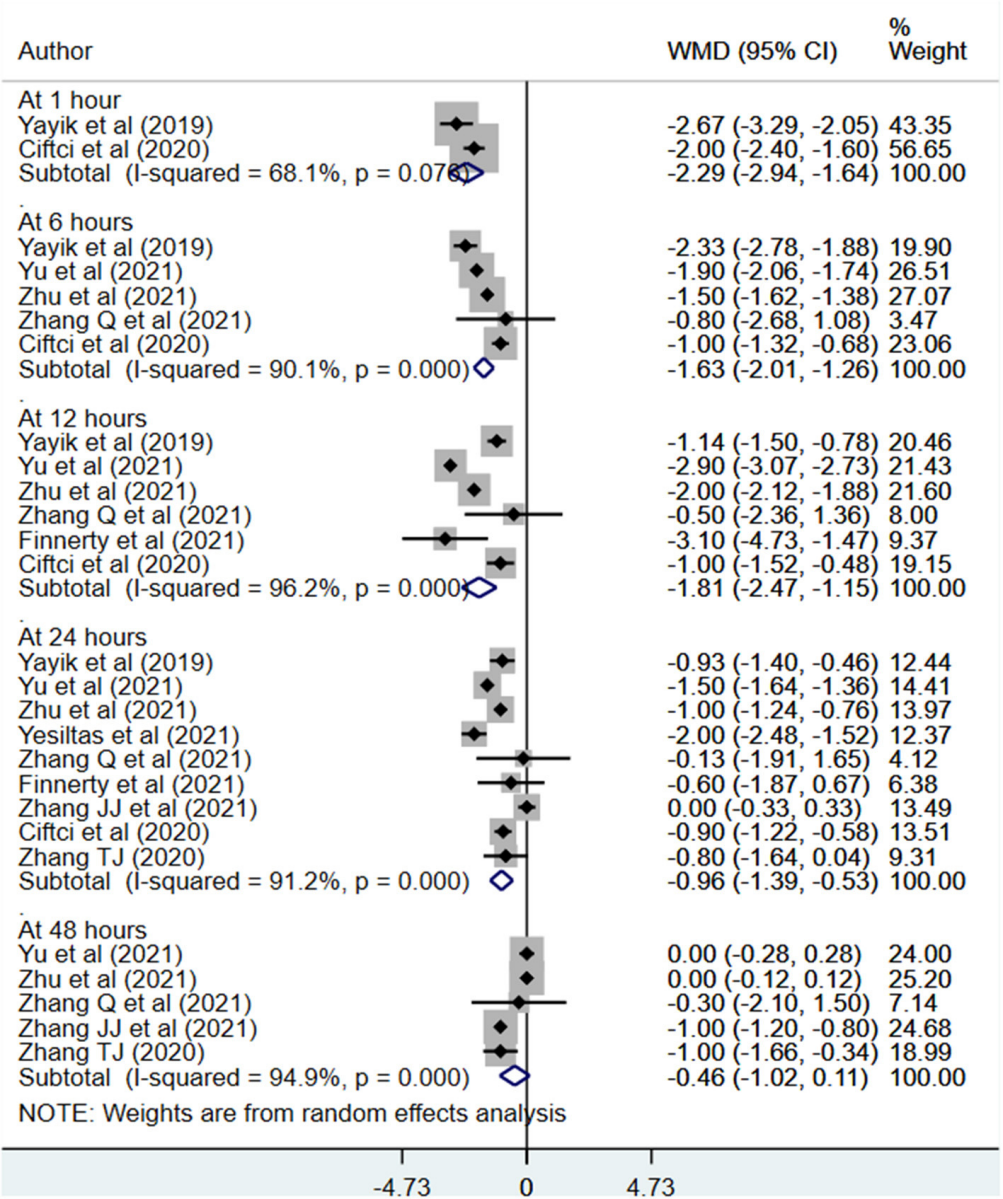
Postoperative pain scores at movement among subjects with erector spinae plane block, compared to placebo or no block.

Patients receiving ESPB reported comparatively lesser pain score at 1 h (WMD −1.62, 95% CI: −2.55, −0.69; *N* = 7, *I*^2^ = 95.7%), 6 h (WMD −1.10, 95% CI: −1.45, −0.75; *N* = 10, *I*^2^ = 91.8%), 12 h (WMD −0.78, 95% CI: −1.23, −0.32; *N* = 11, *I*^2^ = 96.9%) and 24 h (WMD −0.54, 95% CI: −0.83, −0.25; *N* = 13, *I*^2^ = 92.8%) post-operatively compared to those receiving either placebo or no block (Figure 4, Table 3). There was no statistically significant difference in the reported pain scores between patients in both the groups at 48 h post-operatively. Similar findings were noted for pain scores at movement wherein those receiving ESPB reported on comparatively lesser pain score at movement at 1 h (WMD −2.29, 95% CI: −2.94, −1.64; *N* = 2, *I*^2^ = 68.1%), 6 h (WMD −1.63, 95% CI: −2.01, −1.26; *N* = 5, *I*^2^ = 90.1%), 12 h (WMD −1.81, 95% CI: −2.47, −1.15; *N* = 6, *I*^2^ = 96.2%) and 24 h (WMD −0.96, 95% CI: −1.39, −0.53; *N* = 9, *I*^2^ = 91.2%) post-operatively compared to those receiving either placebo or no block (Figure 5, Table 4).

**Table 3:** Summary result of the postoperative pain scores at rest, comparing subjects with erector spinae plane block to those with placebo or no block.

**Time point**	**Pooled effect size (95% confidence interval, CI)**	**Number of studies (no. of participants)**	***I*** **^2^ (%)**
0–1 h	WMD −1.62 (−2.55, −0.69)*	7 (*n =* 451)	95.7
At 6 h	WMD −1.10 (−1.45, −0.75)*	10 (*n =* 631)	91.8
At 12 h	WMD −0.78 (−1.23, −0.32)*	11 (*n =* 691)	96.9
At 24 h	WMD −0.54 (−0.83, −0.25)*	13 (*n =* 810)	92.8
At 48 h	WMD −0.07 (−0.18, 0.04)	7 (*n =* 480)	28.7

* *Statistically significant at P < 0.05*.

**Table 4:** Summary result of the postoperative pain scores at movement, comparing subjects with erector spinae plane block to those with placebo or no block.

**Time point**	**Pooled effect size (95% confidence interval, CI)**	**Number of studies (no. of participants)**	***I*** **^2^ (%)**
0–1 h	WMD −2.29 (−2.94, −1.64)*	2 (*n =* 120)	68.1
At 6 h	WMD −1.63 (−2.01, −1.26)*	5 (*n =* 300)	90.1
At 12 h	WMD −1.81 (−2.47, −1.15)*	6 (*n =* 360)	96.2
At 24 h	WMD −0.96 (−1.39, −0.53)*	9 (*n =* 535)	91.2
At 48 h	WMD −0.46 (−1.02, 0.11)	5 (*n =* 299)	94.9

* *Statistically significant at P < 0.05*.

### Complications and Other Outcomes

**Figure 6:**
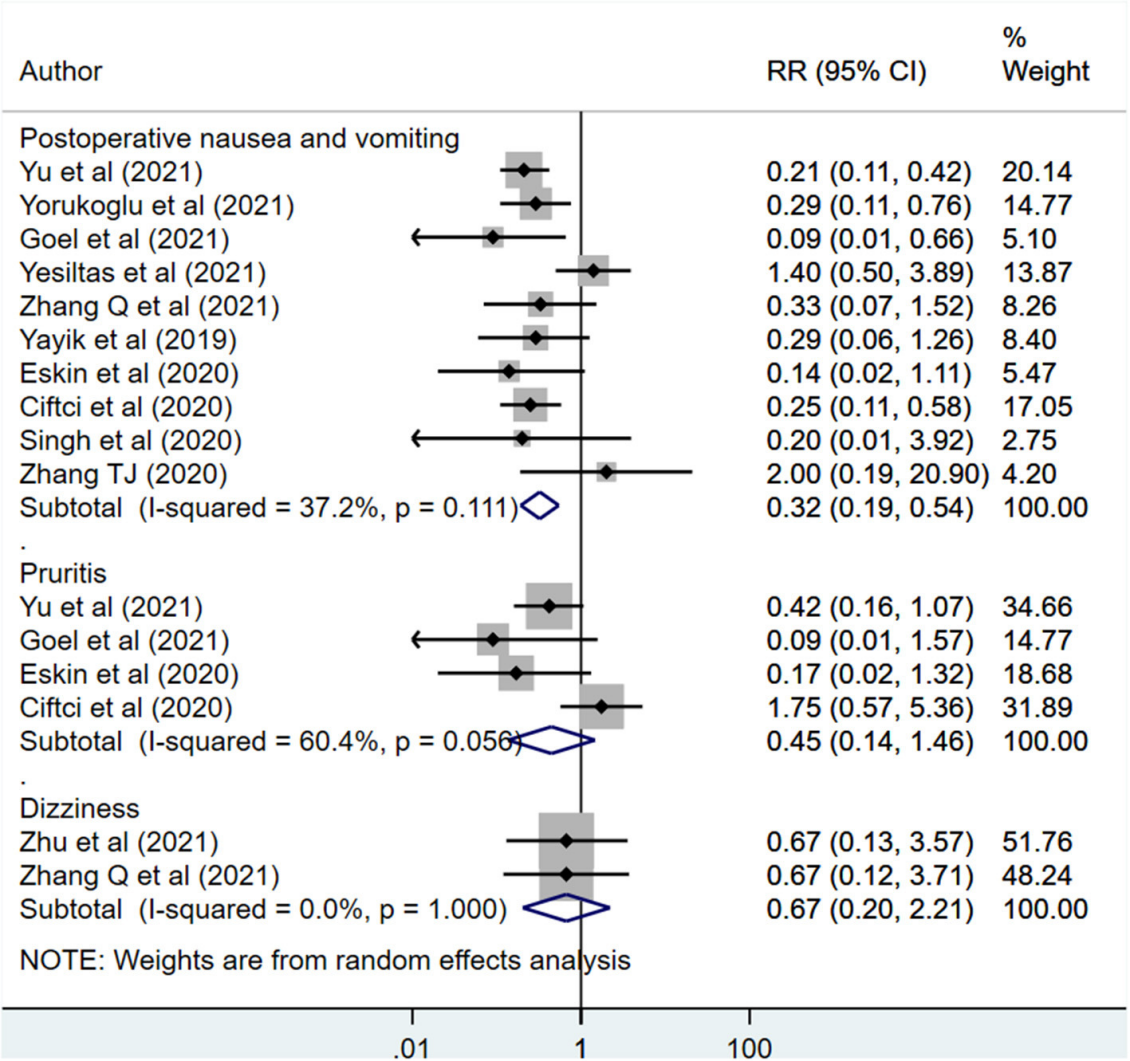
Risk of complications among subjects with erector spinae plane block, compared to placebo or no block.

Postoperative nausea and vomiting (PONV) risk (RR 0.32, 95% CI: 0.19, 0.54; *N* = 10, *I*^2^ = 37.2%) was lower in those receiving ESPB compared to those with placebo/no block (Figure 6). However, pruritis risk (RR 0.45, 95% CI: 0.14, 1.46; *N* = 4, *I*^2^ = 60.4%) and dizziness (RR 0.67, 95% CI: 0.20, 2.21; *N* = 2, *I*^2^ = 0.0%) was similar in both the groups. Egger's test indicated no evidence of publication bias (*P* = 0.22 for PONV, *P* = 0.12 for pruritis and *P* = 0.82 for dizziness). There were no differences in the duration of surgery, intra-operative blood loss, and hospital stay duration between the two groups (Table 2).

## Discussion

ESPB block is induced through injection of local anesthetic between the erector spinae muscle and the transverse process of the vertebrae (9–11). Upon injection, the anesthetic diffuses both cranially and caudally and acts on the dorsal rami of the spinal nerves. Some studies suggest that ESPB, similar to epidural analgesia, may play a dual role of visceral and somatic analgesia (37, 38). One of the advantages of ESPB is that is ultrasound guided and the plane of delivery of the local anesthetic is away from the spinal cord. This minimizes the risk of spinal injury. Further, the risk of hematoma and pneumothorax is minimal with this technique. All this collectively make ESPB an attraction procedure of choice for post-operative analgesia.

The current meta-analysis was conducted to provide updated evidence on the efficacy of ESPB, compared to placebo or no block for patients undergoing spinal surgical procedures. Through pooling of findings from 13 RCTs, the review noted a substantial reduction in total opioid use post-operatively, reduced need for rescue analgesia, need for lower amount of rescue analgesia and had a higher demand time for analgesia. Patients receiving ESPB reported comparatively lesser pain score at 1 to 24 h post-operatively, both at rest and on movement. At 48 h post-operative period, the pain scores were similar in both the groups. Further, the risk of postoperative nausea and vomiting (PONV) was lower in those receiving ESPB. There were no differences in the duration of surgery, intra-operative blood loss and length of hospital stay between the two groups. On GRADE assessment, the quality of the pooled findings was low to moderate.

The findings are similar to the previous meta-analysis on this issue. Jun Ma et al. in their review of 12 studies (*n* = 828) documented a significant effect of ESPB on reducing pain scores at rest until 48 h post-operatively but only until 24 h post-operatively on movement (15). There was also a reduced consumption of opioid and a reduced risk of need for rescue analgesia in those that received ESPB. In the ESPB group, there was also a reduced risk of PONV (15). A major limitation of this review is that the authors included non-peer reviewed research findings provided in thesis/dissertation. Further, the review did not provide any evidence on the quality of the pooled findings. Another recent review by Liu et al. documented significantly lower requirement for opioid post-operatively, reduced postoperative pain scores and reduced risk of need for rescue analgesia (16). The risk of postoperative nausea and vomiting was significantly lower in the ESPB group. Kendall et al. in their review included studies among patients undergoing surgical procedures and not necessarily restricted their analyses to only those with spinal surgery (39). They included 13 RCTs with 679 patients and found that ESPB reduced post-operative opioid consumption, as well as post-operative pain at 6 h. This review provided moderate quality evidence in support of ESPB being a useful strategy for alleviation of post-operative pain (39).

Our current review and meta-analysis reaffirm the findings of the previous reviews but does that through better search, identification and inclusion of all relevant publications. The findings of our analysis have important clinical implications as the post-operative pain following spinal surgery affects a substantial proportion of patients (around half) and insufficient pain control may probably delay attainment of early mobilization and rehabilitation. This, further, could lead to sub-optimal well-being of the subjects and could culminate into persistent post-operative pain that may require prolonged use of pain medications. We also did the GRADE assessment of the pooled findings and noted a low to moderate quality for most of the outcomes. Low to moderate quality indicates that further research is required and is likely or very likely to have an important impact on the confidence in the estimate of effect and may change the estimate.

There are some of the limitations of this meta-analysis. In some of the studies, double blinding was not done. Inclusion of such articles may adversely impact the quality of the evidence. The sample sizes considered in the included studies was small. Future studies may be done with larger sample sizes and robust methodology. One of the key limitations is that we were unable to examine the dose response effect of ESPB owing to the lack of substantial variability in the dose of the anesthetic used. For some of the outcomes, there was substantial heterogeneity and therefore, we used the random effects model in such instances. This heterogeneity could be due to the variability in the management protocols and skills of treating surgeon across different institutions where the studies were conducted. There could be other factors leading to heterogeneity such as dosage and nature of anesthetic used, patient characteristics, tools used to assess post-operative pain, difference in the nature of analgesics used for management of post-operative pain. Future studies should be conducted with harmonized guidelines for application of ESPB, protocols for management of post-operative pain and measurement of outcomes.

In conclusion, the current meta-analysis suggests that in patients undergoing spinal surgery, ESPB is efficacious, compared to no block/placebo, in reducing pain both at rest and on movement until 24 h post-operatively, total opioid requirement post-operatively, lowering the risk of need for rescue analgesic and the amount of analgesic required. Further, the block reduces the risk of PONV. Future studies should attempt to understand the dose response effect of ESPB on post-operative analgesia and other outcomes.

## Data Availability Statement

The original contributions presented in the study are included in the article/Supplementary Materials, further inquiries can be directed to the corresponding author/s.

## Author Contributions

MD and YX designed the project and prepared the manuscript. MD, YX, and QF were involved in data collection and data analysis. QF edited the manuscript. All authors read and approved the final manuscript.

## Conflict of Interest

The authors declare that the research was conducted in the absence of any commercial or financial relationships that could be construed as a potential conflict of interest.

## Publisher's Note

All claims expressed in this article are solely those of the authors and do not necessarily represent those of their affiliated organizations, or those of the publisher, the editors and the reviewers. Any product that may be evaluated in this article, or claim that may be made by its manufacturer, is not guaranteed or endorsed by the publisher.
