# Supplementary material for: Efficacy of Erector Spinae Nerve Block for Pain Control After Spinal Surgeries: An Updated Systematic Review and Meta-Analysis
Source: Front Surg. 2022 Feb 28;9:845125. doi: 10.3389/fsurg.2022.845125 (PMC8918538; doi:10.3389/fsurg.2022.845125)
Supplement: Supplementary file 1 [file Data_Sheet_1.doc]

**Supplemental table 1. Search strategy for identification of studies to be included in the review**

| **Search strategy**  #1 (erector spinae plane block OR ESPB OR regional anaesthesia OR postoperative analgesia)  #2 (spine surgery OR lumbar surgery)  #3 (outcomes OR pain score OR opioid use OR rescue analgesia OR complications)  #4 (#1 AND #2 AND #3)  #5 (Addresses[ptyp] OR Autobiography[ptyp] OR Bibliography[ptyp] OR Biography[ptyp] OR pubmed books[filter] OR Case Reports[ptyp] OR Congresses[ptyp] OR Consensus Development Conference[ptyp] OR Directory[ptyp] OR Duplicate Publication[ptyp] OR Editorial[ptyp] OR Systematic reviews OR Meta analysis OR Festschrift[ptyp] OR Guideline[ptyp] OR In Vitro[ptyp] OR Interview[ptyp] OR Lectures [ptyp] OR Legal Cases[ptyp] OR News[ptyp] OR Newspaper Article[ptyp] OR Personal Narratives [ptyp] OR Portraits[ptyp] OR Retracted Publication[ ptyp] OR Twin Study[ptyp] OR Video-Audio Media[ptyp])  #6 (#4 NOT #5) |
| --- |

**Supplementary table 2. Author’s judgements about each risk of bias for each included study based on Cochrane risk of bias assessment items**

| **Included studies** | **Random sequence generation** | **Allocation concealment** | **Blinding of participants and personnel** | **Blinding of outcome assessment** | **Attrition bias** | **Other bias** |
| --- | --- | --- | --- | --- | --- | --- |
| Yu et al (2021) | + | + | ? | + | - | - |
| Zhu et al (2021) | + | + | + | + | - | - |
| Yorukoglu et al (2021) | + | + | + | + | ? | - |
| Goel et al (2021) | + | + | + | + | - | - |
| Yesiltas et al (2021) | + | + | + | + | - | - |
| Zhang Q et al (2021) | + | + | + | + | ? | - |
| Finnerty et al (2021) | + | + | ? | + | - | - |
| Zhang JJ et al (2021) | + | + | ? | + | - | - |
| Yayik et al (2019) | + | ? | - | + | - | - |
| Eskin et al (2020) | + | + | - | + | ? | - |
| Ciftci et al (2020) | + | ? | - | + | - | - |
| Singh et al (2020) | + | ? | - | + | - | - |
| Zhang TJ et al (2020) | + | + | + | + | ? | - |

+ denotes presence; ? denotes unclear; - denotes absence

**Supplementary Table 3. Quality of evidence according to the GRADE criteria**

| **Outcome** | **Pooled effect size** | **Number of studies (Design)** | **Characteristics of included studies** | | | | |  |
| --- | --- | --- | --- | --- | --- | --- | --- | --- |
|  |  |  | **Risk of bias** | **Inconsistency** | **Indirectness** | **Imprecision** | **Publication bias** | **Overall GRADE quality score** |
| Total opioid use | SMD -2.76 (95% CI: -3.69, -1.82); I^2^=95.7% | N=12 (all RCTs) | Not serious | Serious | Serious | Not serious | Undetected | ⨁⨁◯◯ Low |
| Need for rescue analgesia | RR 0.38 (95% CI: 0.22, 0.66); I^2^=85.1% | N=8 (all RCTs) | Not serious | Not serious | Serious | Not serious | Undetected | ⨁⨁⨁◯ MODERATE |
| Dose of rescue analgesic | SMD -5.08 (95% CI: -7.95, -2.21); I^2^=98.5% | N=5 (all RCTs) | Not serious | Serious | Serious | Not serious | Undetected | ⨁⨁◯◯ Low |
| Post-operative nausea and vomiting | RR 0.32 (95% CI: 0.19, 0.54); I^2^=37.2% | N=10 (all RCTs) | Not serious | Not serious | Serious | Not serious | Undetected | ⨁⨁⨁◯ MODERATE |
| Post-operative pruritis | RR 0.45 (95% CI: 0.14, 1.46); I^2^=60.4% | N=4 (all RCTs) | Not serious | Not Serious | Serious | Serious | Undetected | ⨁⨁◯◯ Low |
| Post-operative dizziness | RR 0.67 (95% CI: 0.20, 2.21); I^2^=0.0% | N=2 (all RCTs) | Not serious | Not Serious | Serious | Serious | Undetected | ⨁⨁◯◯ Low |
| Post-operative pain score at 1 hr | WMD -1.62 (95% CI: -2.55, -0.69); I^2^=95.7% | N=7 (all RCTs) | Not serious | Serious | Serious | Not serious | Undetected | ⨁⨁◯◯ Low |
| Post-operative pain score at 6 hr | WMD -1.10 (95% CI: -1.45, -0.75); I^2^=91.8% | N=10 (all RCTs) | Not serious | Serious | Serious | Not serious | Undetected | ⨁⨁◯◯ Low |
| Post-operative pain score at 12 hr | WMD -0.78 (95% CI: -1.23, -0.32); I^2^=96.9% | N=11 (all RCTs) | Not serious | Serious | Serious | Not serious | Undetected | ⨁⨁◯◯ Low |
| Post-operative pain score at 24 hr | WMD -0.54 (95% CI: -0.83, -0.25); I^2^=92.8% | N=13 (all RCTs) | Not serious | Serious | Serious | Not serious | Undetected | ⨁⨁◯◯ Low |
| Post-operative pain score at 48 hr | WMD -0.07 (95% CI: -0.18, 0.04); I^2^=28.7% | N=7 (all RCTs) | Not serious | Not serious | Serious | Not serious | Undetected | ⨁⨁⨁◯ MODERATE |
